# Intensive Psychotherapy Training in Korean Psychiatric Residency Programs

**DOI:** 10.4306/pi.2008.5.4.221

**Published:** 2008-12-31

**Authors:** Sang Min Lee, Geon Ho Bahn, Won Hae Lee, Jae Jin Lee, Seo Kyung Lee, Jin Kyung Park, Sang Bin Paik

**Affiliations:** 1 Department of Neuropsychiatry, Kyung Hee University, School of Medicine, Seoul, Korea.; 2 Department of Psychiatry, Kyung Hee University East-West Medical Center, Seoul, Koera.; 3 Department of Psychiatry, University of Ulsan College of Medicine, Asan Hospital, Gangneung, Korea.

**Keywords:** Intensive psychotherapy, Supervision, Resident training

## Abstract

**Objective:**

The authors investigated the current practice of intensive psychotherapy by residents in the department of psychiatry.

**Methods:**

We mailed a questionnaire to 126 fourth-year psychiatry residents in order to obtain data on their clients' sociodemographic characteristics, the settings in which psychotherapy is being conducted, the effects of psychotherapy, the difficulties associated with psychotherapy, the state of supervision and the level of clients' satisfaction.

**Results:**

Approximately 51.5% of the residents completed the questionnaires. The average number of clients was 4.9±3.8, the average number of psychotherapy sessions was 26.2±20.1, and 69.4% of the residents had performed insight-oriented psychotherapy. Approximately 69.8% of the fourth-year residents had received some form of supervision, and 58.7% agreed to increase the frequency of supervision. Approximately 74.2% of the cases were supervised. The average number of supervisions per case was 9.2±10.5.

**Conclusion:**

The setting in which psychotherapy is conducted, number of clients, and type of supervision varied greatly among the training institutes surveyed. Based on these findings, we expect to create better psychotherapy training programs for psychiatric residents.

## Introduction

The introduction of modern Western psychotherapy in Korea began immediately after the Korean War.^1^ There have been some difficulties associated with its application in the Korean society, but it is now commonly accepted as an important psychiatric therapeutic procedure. Although the rapid development of psychopharmacology resulted in a relative reduction in the number of patients receiving psychotherapy, the demand for psychotherapy is increasing due to the improvement of Korea's socioeconomic status.^2^ During the procedure, each educational hospital made greater efforts to intensify the education and training of their residents in psychotherapeutic techniques, and an academic meeting for the specialization of psychotherapy began to form. Since the 1970s, psychiatric residents have been subjected to substantial amounts of supervision while practicing psychotherapy,^3^ and residents are now required to conduct psychotherapy during the resident training period in order to become certified psychiatrists. In addition, the performance record of psychotherapy has been systemized to allow for evaluation. Therefore, there has been increasing interest in determining how well Korean psychiatry residents actually understand psychotherapy and how the therapy is conducted. There are a few similar reports concerning the review of psychotherapy by residents, analysis of the current status of supervision,^4^ experience of resident instruction from the perspective of supervisors^5^,^6^ and the experience of residents and supervisors who conducted the psychotherapy group supervision.^7^

Although psychiatry has rapidly developed in various fields in recent years, the importance of psychotherapy in psychiatry has been proven in numerous studies. Mohl et al.^2^ stressed the importance of recognizing that education in psychotherapy is the fundamental and most important factor in becoming a talented psychiatrist, even with the changing of the times. A balanced resident training program in the field of psychotherapy is now more important than ever before. In addition, supervision is an inevitable component for proper psychotherapy training because it affects the quality of improvements by the therapist and treatment outcomes by reviewing the interactions between the therapist and the patient. Even though the numbers of residents have continuously increased with the increase in the number of psychiatry training hospitals, there are not enough programs for properly educating residents in psychotherapy through education analysis and supervision under the current situation. The realities that much value has been placed on biological therapy, while little value was given to psychotherapy during psychiatric residency training in the U.S were very concerning. Investigations into the situation have taken place since the early 1990s, and since the 2000s, the committee has been organized, and progress has been made in discussions concerning the amount and method of psychotherapy required for a well-balanced psychiatric training program.^8^ However, in Korea, there has been no research concerning the current status of psychotherapy training since the early 1990s.

Therefore, the authors investigated how psychiatric residents are conducting psychotherapy and how they are being supervised. Due to the lack of domestic investigation data regarding these issues, the authors surveyed the current situation and intended to reflect the study result in the establishment of a resident training policy after comparing the current result with those of previous studies.

## Methods

### Subjects

The current study collected data by mailed the final version of a questionnaire to 126 fourth-year residents working at 72 training hospitals around the nation between June and July 2007. Sixty-five (51.5%) of the 126 residents responded to the survey. Among the respondents, 85% worked at university hospitals, and 8.5% and 6.3% of them were training in hospitals specializing in psychiatry and general hospitals, respectively. The non-respondents were called, sent mailings and e-mailed repeatedly up to three times. Anyone who did not respond the questionnaires after the third try was defined as a 'rejecter'. The rejection rates were 46% for University hospitals, 14.2% for general hospitals and 73.6% for hospitals specializing in psychiatry, respectively. The rejection rates in each region were 43.4% in Seoul, 49.7% in Gyeonggi, 71.5% in Gangwon, 58.4% in Chungcheng, 58.4% in Gyeongsang, and 44.5% in Jeolla.

### The questionnaire

A senior researcher and collaborative researchers held a preliminary meeting to prepare the first questionnaire after reviewing the previously reported data on the status of psychotherapy and investigating the related questionnaires. The first survey was conducted on five fourth-year residents in psychiatry by using the first preparation, and problems were dealt with in order to develop the final version of the questionnaire. The questionnaire asked about the following: 1) Sociodemographic and clinical characteristics of clients, including the clients' sex, age, education level, socioeconomic status, and diagnosis; 2) Clinical variables of clients, including the start of therapy, types of psychotherapy practiced, frequency of preliminary interviews, total frequency, whether or not therapy was terminated, situation at the time of therapy termination, causes of therapy termination after negotiation, reason for stopping therapy, important difficulties faced at the time of psychotherapy, combination of drug therapy or not, major substance of drug therapy, route of asking in psychotherapy, and effects of psychotherapy; 3) the settings in which psychotherapy was conducting, including weekly frequency of psychotherapy, estimation of psychotherapy fee, time point at which the fee for psychotherapy is paid, fee when a client does not arrive on time, whether or not the psychotherapy is recorded, and whether or not the psychotherapy is memorized; and 4) the supervision of psychotherapy, including the method of supervision, place of supervision, and frequency of supervision and level of client satisfaction. The type of psychotherapy used during the study was limited to intensive psychotherapy, which includes analytical psychotherapy and supportive psychotherapy. Cognitive-behavioral therapy was not included in this study.

### Statistics

Among several variables, continuous variables were calculated to obtain mean values and standard deviations, and frequency analyses were performed for nominal scales. Analysis of variance (ANOVA) was used for comparisons among the investigated items. The nominal scales were analyzed using cross analysis. Statistical analyses of all evaluation data were performed using the SPSS 13.0 (SPSS Inc., Chicago, USA) program operated in Windows.

## Results

### Characteristics of clients receiving psychotherapy

Each resident treated 4.9±3.8 psychotherapy clients (ranging from one to 22 cases) during their resident training.

The mean age of the clients was 28.4±8.5 years, and most of the clients were female (66.7%). Investigations into the education levels of the clients indicated that 39.6% of them graduated from high school, and 51.7% of them had a degree equal to or beyond a college degree. The distribution of socioeconomic status from the most prevalent to the least was III, II, IV, I, and V. Socioeconomic status was higher than Group III in 79% of the total clients. Cases diagnosed with Axis-I disorders comprised 40.8% of the clients, while 6.6% of the clients had Axis-II diagnoses. The rest of the cases had both Axis-I and Axis-II diagnoses. Depressive disorder, anxiety disorder, and adjustment disorder were frequently found in clients with Axis-I diagnoses. In the clients with Axis-II diagnoses, 2.1% were cluster A, 72.5% were cluster B, and 22.5% were cluster C as demonstrated by the prevalence of borderline personality disorder, followed by histrionic personality disorder, and avoidant personality disorder.

### Characteristics of psychotherapy cases (Table 1)

Although 2.1% of the respondents said they began studying psychotherapy during the first semester of their first year of resident training, 23% responded that the start of psychotherapy was in the first semester of the second year of resident training, and 24.6% responded that the start of psychotherapy during the first semester of their third year of resident training, thus showing a gradually increasing trend as the year of resident training increased.

When asked about the type of psychotherapy practiced, 67.4% responded by saying they practiced insight-oriented psychotherapy, 28.5% reported practicing supportive psychotherapy, and 4.2% report practicing psychoanalysis. In the initial stages of psychotherapy, 69.5% of the clients receiving the psychotherapy were outpatients, and 30.5% received the treatment during hospitalization.

Regarding the means by which psychotherapy was requested, 39.9% of the cases were requested by outpatients visiting physicians, 28.1% were requested by other physicians at the same hospital, 26.3% were requested by the attending physicians of a hospital department, and 5.7% were requested by other hospitals.

The average frequency of preliminary meetings was 2.2±2.7 sessions. The average frequency of psychotherapy was 26.2±20.1 times per case, with a minimum of 1 and a maximum of 150 times. The case in which 150 sessions of psychotherapy were recorded was excluded from the analysis in order to reduce the deviation. For each session of psychotherapy, the average treatment time was 47.8±5.5 minutes.

Psychotherapy was terminated in 71.7% of the cases. In most cases (57.9%), the psychotherapy was terminated due to the client's failure to show up for therapy. The reasons for discontinuing therapy were failure to overcome transference resistance in 49.2% of the cases, high expectations for a treatment effect in 13.8%, economical burdens in 6.2%, and noncooperation of guardians in 3.8%.

Successful completion of the therapy was reported by 17% of the respondents. Successful completion of therapy could be attributed to the client's motivation in 51.5% of the cases, psychopathology of the client in 27.3%, and appropriate supervision in 12.1%. The greatest difficulty experienced by the resident while conducting psychotherapy was a lack of client understanding, followed by lack of supervision, lack of time, lack of understanding by the client's family, and lack of economical capacity, which showed the trend of highly accounting the causes from the therapists' point of view.

Although psychotherapy was only conducted in 31.7% of the cases, 68.3% of the cases were treated using a combination of drug therapy and psychotherapy, and the drugs used to treat 56.1% of the combined drug therapy cases were prescribed by the physician administering the therapy

### Psychotherapy setting

When asked about the treatment time, 61.3% of the respondents said they conducted the therapy during regular business hours. The average frequency of psychotherapy sessions was 1.1±0.4 times a week. The expenditure for one session differed according to the calculation methods of the training hospital, and the average cost was 28,029±10,007 won (Ca 30±10 US dollars) for each session of therapy. Most of the sessions were paid in full after each treatment. Even if clients did not arrive on time, half of the hospitals did not charge the expenditure at the next session. When conducting psychotherapy, 59.2% of the respondents recorded the session, and 47.5% memorized the therapy.

### Psychotherapy supervision

When asked about the supervision of psychotherapy, 74.2% of respondents reported receiving some form of supervision, and 38.8% reported receiving individual supervision (Table 2). The average frequency of supervision was 9.2±10.5 times, 74.9% of the supervisions were performed within the hospital, and 64.2% of the residents who received the supervision were satisfied with it.

### Attitude of respondents toward psychotherapy

The need to increase the amount of psychoanalysis and psychotherapy conducted during residency training was recognized by 58.7% of the respondents recognized, and 41.3% believed that the current training level is appropriate. None of the respondents believed there was a need to reduce the amount of therapy conducted during the training.

### Comparison between investigated items

In the case of female clients, many cases of psychotherapy were successfully terminated without showing statistical significance, and no difference was observed. Individual supervision was mainly conducted in university and general hospitals, while group supervision was conducted more frequently in hospitals specializing in psychiatry, but this difference was not significant. Third- and fourth-year residents began conducting insight-oriented psychotherapy more frequently than first- and second-year residents (p=0.045). The total duration of treatment was longer when the residents started the therapy during their first or second year of residency (p=0.042), and the frequency of receiving supervision was also high (p=0.026), but no statistically significant difference was found when comparing the cases in which therapy was terminated. The difference was thought to be attributable to numerous cases of currently progressing psychotherapies for third- and fourth-year residents.

## Discussion

Consistent with the study by Stirman et al.,^9^ which reported that 58% of outpatients who received psychotherapy were initially diagnosed with adjustment disorder or dysthymic disorder, the results of the current study also indicated the prevalence of depressive disorder, anxiety disorder, and adjustment disorder among the clients with Axis-I diagnoses. This could be attributable to an increase in the number of individuals who had difficulty adjusting to their current situation due to the advancement and specialization of the society, which correspondingly increased the likelihood that they would visit a psychiatrist.

In clients with Axis-II diagnoses, most of the psychotherapies were performed in clients with cluster B characteristics, and borderline personality disorder was the most prevalent disorder, comprising 39.4% of the single diagnoses. This finding was similar to that published by Rhee and Rhee,^10^ who reported that 41% of clients receiving psychotherapy alone had borderline personality disorder and that there were twice as many females as males.

Many of the residents performed psychotherapy during their second and third years of residency training, and the proportion of residents starting psychotherapy for hospitalized clients were found to be low, at just 30.5%. This is considered to be attributable to the fact that first-year residents are mainly responsible for the treatment of hospitalized clients, and residents in the later years of training usually see outpatients and clients with personality disorders. However, because the continuation of psychotherapy training is circumstantially difficult after finishing the residency program, it is desirable to start the psychotherapies during the early years of the resident training program in order to experience long-term progression of psychotherapy. This is related to the direction of training provision suggested by the Korean Neuropsychiatric Association. The resident's psychotherapy evaluation categories listed on the resident training regulation status evaluation form^11^ (revised on April, 2001) developed by the Korean Neuropsychiatric Association are defined as follows. In the evaluation category of diagnosis, the in-depth psychotherapy part assigns a full score of 10 when residents past the second year of training conduct psychotherapy more than 10 times, regardless of the case. The supportive psychotherapy part assigns a full score when residents past the second year of training treat more than 5 cases, and the psychotherapy individual supervision and group supervision items can be assigned full scores if they are regularly conducted. In other words, it could be interpreted as indicative of the fact that first-year residents do not need to conduct psychotherapy and are satisfied with only a single case of in-depth psychotherapy, or it could be interpreted as indicative of the fact that the first-year residents could not conduct psychotherapy.

The quality and evaluation of the effect of psychotherapy during the supervision of a psychotherapy session are uncertain. The result obtained in the current study could be considered to reflect such a general level of evaluation categories. Although ten residents selected psychoanalysis as a type of psychotherapy practiced, the frequency at which they actually conducted psychotherapy was limited to once a week. Therefore, none of the respondents were conducting psychoanalysis in their regular practice.

Regarding the frequency of therapy, there was no case requiring more than three sessions a week. Considering that a lack of time was deemed to be one of the main difficulties associated with psychotherapy, the heavy workloads given to residents could be considered to be a large obstacle in the performance of psychotherapy.

In the current study, residents reported treating a client for about three to seven months. Previous studies^12^-^14^ on the appropriate number of psychotherapy sessions and length of the treatment period indicated that increasing the frequency of therapy to more than two times a week and extending the therapy period could lead to better continuous effects. In the future, it is considered that the training direction of residents should be guided to allow residents to have more long-term experiences with indepth psychotherapy during their training.

In a study conducted by Kang^15^ in 1978, 55.1% of psychotherapies conducted by residents were requested by professors for outpatients, and only 3.8% were conducted based on the therapist's decision alone. However, the proportion of therapists working as outpatient attending physicians or as attending physicians in a hospital in the current study was found to be increased up to 66.2%. However, the number of cases in which psychotherapy was requested by other physicians at the same hospital were reduced to 28%. Such changes indicate that residents are currently carrying out psychotherapies in voluntary and active manners and that the psychotherapies conducted by residents are now more popular than ever before.

In a study by Rhee and Yoon,^3^ the client's motivation and client's psychopathology were suggested to be the most important variables for successful therapy termination, and the results of the current study were consistent with those findings. Although the most important factor in terminating therapy was cited as the frustrated expectations of clients seeking magical effects of therapy, the current study found that 49.2% considered the failure of coping transference resistance to be the most important variable for failure, and only 13.8% of the cases did not satisfy the high expectation result by differing from conventional studies. The client's lack of economic capacity was reported to be the greatest source of difficulty while conducting psychotherapy in a previous study.^3^ However, in the present study, the respondents reported that a lack of client understanding was the greatest source of difficulty while conducting psychotherapy, and a lack of economic capacity was the least-mentioned cause. This indicates the need to conduct an in-depth educational program on psychodynamics and provide supervision when residents have psychotherapy sessions.

The clinical effects of psychodynamic psychotherapy have already been proven, and it is known to be as effective as cognitive behavioral therapy in patients with specific psychiatric diseases.^16^ Approximately 75.5% of the residents who participated in the current study also responded that the therapy was effective, but few of them reported having successful termination of therapy. This is an important difficulty in the practice of psychotherapy, and it corresponds to the answers that place much importance on the causes of difficulty experienced by therapists.

The importance of supervision during psychotherapy training has been emphasized with educational analysis.^7^ Both individual and group supervision have been used. Although individual supervision is the optimal approach, these two types of approaches have been used in combined forms at domestic resident training hospitals due to circumstantial causes, including lack of supervisors and social conditions.^1^ The current study results revealed an increase in the proportion of residents receiving supervision and a high level of satisfaction among residents receiving supervision. However, the proportion of residents receiving individual supervision was low, and many cases of supervisions in hospitals specializing psychiatry were done through group supervision, resulting in a greater number of cases receiving group supervision than in the university or general hospital setting, which highlighted the need for more individual supervision. The results of the current study seem to be meaningful given the current situation, as the discussion as to how to handle the field of psychotherapy on an in-depth scale in resident training is currently underway.

Although it has been expected to be related to total therapy hours, therapy type, therapy success rate, cost of psychotherapy, and supervision frequency as the therapy time is delayed, no such relationship was reported in actual circumstances. However, it is thought that this result alone could not be used to indicate a lack of correlation between the psychiatric training period and psychotherapy skill. It is therefore necessary to establish an evidence-based training system in order to determine whether the start of psychotherapy training during residency is indeed appropriate. If so, the year that resident training has to be started, the number of cases that have to be experienced, and how the supervisor training itself has to be managed must be established.

The current study has some limitations. One limitation of this study is that it only included fourth-year residents. In addition, since most of the respondents had been training at university hospitals, it was difficult to draw any conclusion that would be representative of the entire population of psychiatric residents. Probably due to the complexity of the questionnaires, the reply rate was only limited to 51.5%, even though the residents were contacted three times and encouraged to participate. The possibility of that some of the residents skipped their education in psychotherapy cannot be excluded. Since the current investigation was conducted in the form of a survey, the limitation of questions and the issue of reliability should be also considered. For instance, some respondents reported conducting psychoanalysis, even though they only conducted psychotherapy. The reported rate at which psychotherapy was conducted in inpatients was 30.5%, which was far from reality. Furthermore, although about half of the treatment failure was due to unsolved transference resistance, these conclusions were based on the interpretation of therapists alone (if they did not receive supervision). There was a difference between the total number of clients receiving psychotherapy and the number of psychotherapy clients analyzed, which might be have resulted from respondents failing to report all of the psychotherapy cases they treated due to the large amount of time required to prepare the data.

Despite the above limitations, the current study could find meaning from its systematic attempt to determine the status of intensive psychotherapy in psychiatric residents by conducting a survey. The objective of psychotherapy education has been stated as being to cultivate basic grounds for the understanding and practice of psychotherapy, and not to create experts.^8^ The American Psychiatric Association suggested the approaches for cultivating the capacities for the five forms of psychotherapies (Short-term, Cognitive behavior, Combination of drug therapy, Psychodynamics, and Supportive) for residents in training programs from the perspective of the Association in 2000.^17^ It is thought that the current study could be helpful at a time when the work needed to cultivate the substantialities is necessary along with the efforts to increase the frequency of supervision of psychotherapy sessions conducted during the resident training period. In the future, additional studies must be conducted by including residents in all years of training as well as specialists.

In summary, the current study investigated the status of intensive psychotherapy training by conducting a nationwide survey of current fourth-year residents in psychiatry. The results of the study showed that psychiatric residents experienced various types of cases but needed in-depth educational programs concerning psychodynamics as well as intensive supervision. Investigating and analyzing the current status of psychotherapy conducted during the resident training programs in psychiatry will provide fundamental data for establishing a better training environment.

## Figures and Tables

**TABLE 1:**
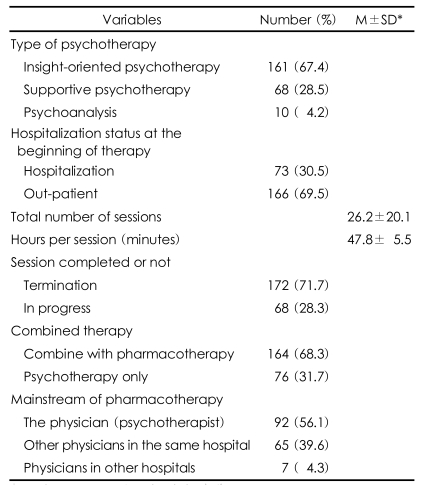
Clinical variables of clients

^*^M±SD: mean±standard deviation

**TABLE 2:**
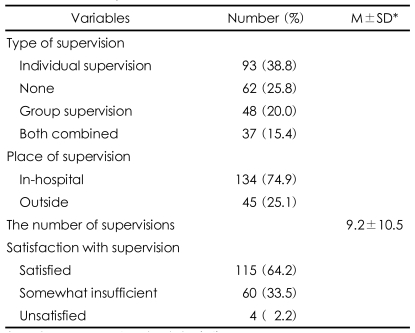
The supervision of psychotherapy

^*^M±SD: mean±standard deviation
